# Patterns in longitudinal growth of refraction in Southern Chinese children: cluster and principal component analysis

**DOI:** 10.1038/srep37636

**Published:** 2016-11-22

**Authors:** Yanxian Chen, Billy Heung Wing Chang, Xiaohu Ding, Mingguang He

**Affiliations:** 1 State Key Laboratory of Ophthalmology, Zhongshan Ophthalmic Center, Sun Yat-sen University, Guangzhou, China; 2 Centre for Eye Research Australia, University of Melbourne, Royal Victorian Eye and Ear Hospital, East Melbourne, Victoria, Australia

## Abstract

In the present study we attempt to use hypothesis-independent analysis in investigating the patterns in refraction growth in Chinese children, and to explore the possible risk factors affecting the different components of progression, as defined by Principal Component Analysis (PCA). A total of 637 first-born twins in Guangzhou Twin Eye Study with 6-year annual visits (baseline age 7–15 years) were available in the analysis. Cluster 1 to 3 were classified after a partitioning clustering, representing stable, slow and fast progressing groups of refraction respectively. Baseline age and refraction, paternal refraction, maternal refraction and proportion of two myopic parents showed significant differences across the three groups. Three major components of progression were extracted using PCA: “Average refraction”, “Acceleration” and the combination of “Myopia stabilization” and “Late onset of refraction progress”. In regression models, younger children with more severe myopia were associated with larger “Acceleration”. The risk factors of “Acceleration” included change of height and weight, near work, and parental myopia, while female gender, change of height and weight were associated with “Stabilization”, and increased outdoor time was related to “Late onset of refraction progress”. We therefore concluded that genetic and environmental risk factors have different impacts on patterns of refraction progression.

Myopia is a common condition worldwide, and has become a major health problem not only by increasingly financial burden of disease, but also for the potential progression towards high myopia leading to irreversible visual impairment^1^,^2^. Evidence of myopia progression has been documented in many studies. An annual progression rate of −0.39 D to −0.68 D was reported in school-age children^3^,^4^,^5^. French *et al*. found that the change in refraction in children with East Asian ethnicity reached up to −1.72 D per year^6^. The striking progression rate of myopia development is a major obstacle to the prevention and treatment of myopia.

Some studies have suggested that the progression of refraction differs between myopes and non-myopes^7^,^8^,^9^. Factors such as parental myopia and near work, were reported to be associated with myopia progression^10^,^11^. However, most of the population-based studies to date have explored the progression of myopia only by calculating annual progression rates as outcome measurements. At present, information about the variety of processes involved in refraction development is still limited, as well as the key determinants in different phases of progression.

Various functions of refraction progression patterns have been proposed since the last century^12^,^13^,^14^,^15^, and this continues to be a topic of interest. Recently, Thorn *et al*. suggested a double exponential growth function fitting myopia progression^16^. The COMET group investigated the myopia stabilization age based on Thorn’s theory using Gompertz curves. All these findings have produced interesting hypotheses of myopia progression patterns and potentially contributing parameters, but are limited to relying on predefined mathematical growth functions.

Cluster analysis is a method of combining data with similar properties, e.g. progression patterns^17^, and is widely used in biology and medical classifications^18^. Principal component analysis (PCA) allows us to extract the main differences in how the refraction evolves over time. Both methods are hypothesis-independent, avoiding the pre-existing bias of traditional model fitting. Harold Saunders attempted to assess the progression of high myopia using clustering, but did not describe the myopia progression patterns in his analysis^19^. Beyond this study, further attempts to estimate myopia progression patterns using cluster analysis and PCA are absent from the literature, to the best of our knowledge.

This paper aims to specifically investigate the different patterns of refraction growth processes in Chinese children using hypothesis-independent methods, and to explore the associations between myopia-related risk factors for each progression component as defined by PCA.

## Results

Table 1 summarized the basic characteristics of the first-born twins. The sample was consisted of 310 boys ad 327 girls, with a mean age at baseline of 10.66 years (SD 2.3). The mean SE in the right eye at baseline was −0.52 D (SD 1.97).

**Figure 1:**
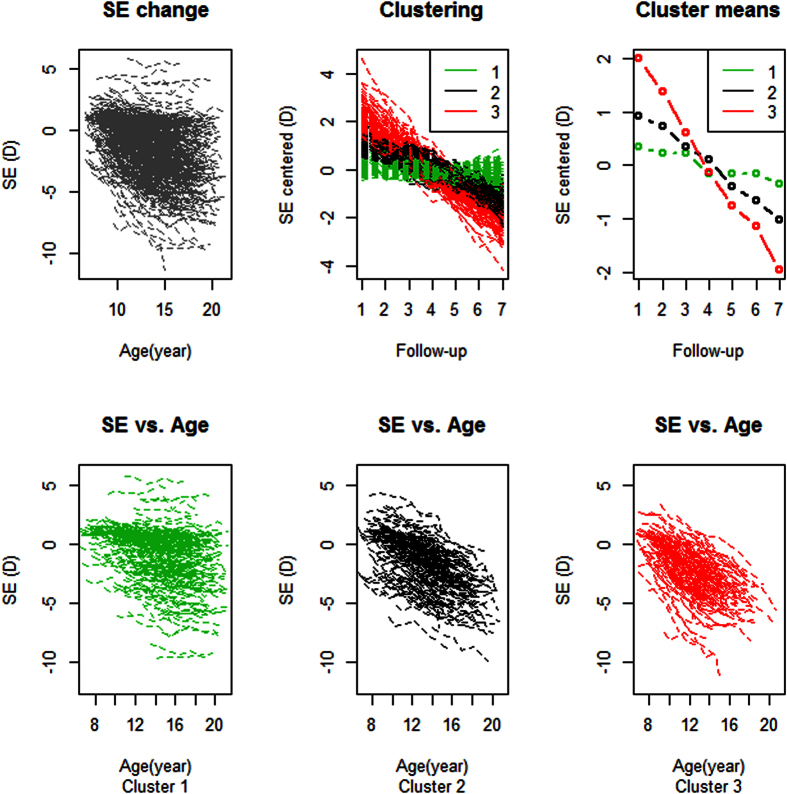
Partitioning clustering of longitudinal refraction in first-born twins. Upper left: refraction change over age in right eyes of all subjects. Upper middle: three clusters of progressing pattern. Upper right: The medoids in the three clusters. Lower row shows that the distributions of cluster 1 to 3 with age respectively.

As shown in Fig. 1, all subjects were classified into three groups with a partitioning clustering analysis according to different refraction progression. Cluster 1 was characterized as progressing steadily over time with the slowest growing slope, while cluster 3 was a group rapidly growing towards a higher level of myopia on the steepest slope. The annual progression rates of clusters 1 to 3 were −0.08 ± 0.08 D, −0.31 ± 0.07 D and −0.58 ± 0.13 D respectively. When comparing cluster 1 with 2, cluster 3 appeared to commence with negative baseline refraction, and a relatively younger age.

Comparisons of demographics, genetic factors, environmental factors and anthropometrics among the three clusters are shown in Table 2. There was an increase in the mean age with increasing growth in gradient (p < 0.0001). After adjusting for baseline age, the mean baseline refraction in cluster 2 was more myopic, with statistical significance (p = 0.0002). Paternal refraction, maternal refraction and proportion of having two myopic parents were also significantly different across the three cluster groups (all p < 0.05).

**Figure 2:**
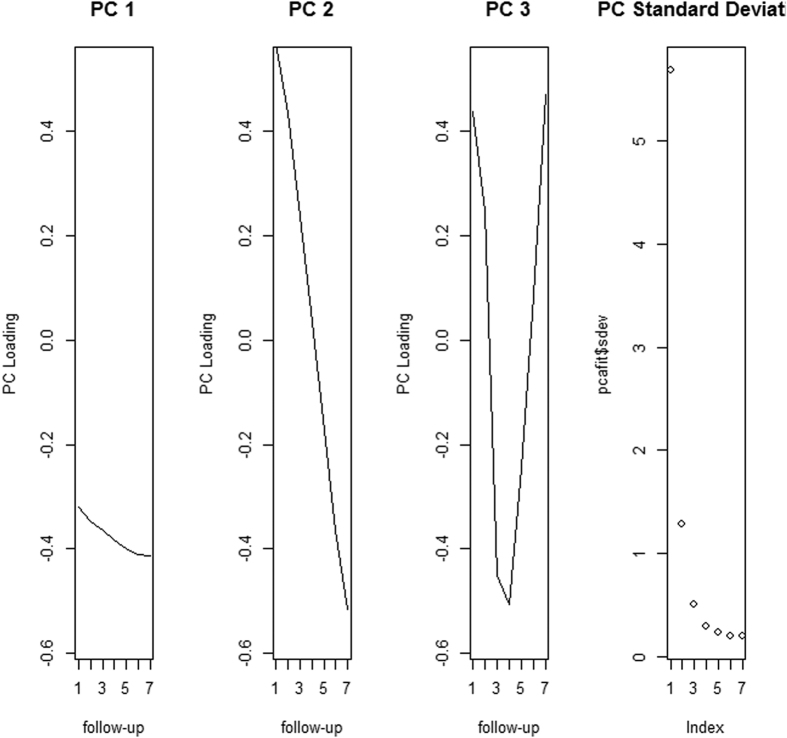
Component loadings plots of the first three principal components of longitudinal patterns in refraction.

There were 7 components of the refraction growth process revealed by PCA. The loadings of components 1 to 3 are plotted against visits in Fig. 2. Component 1, stayed steadily across visits, represented an overall similar level of SE in 7 visits, while component 2 with rapidly changed slope represented the more progressive type of myopia. Component 3 initially decreased, then subsequently increased, indicating a turn occurring at the last few visits. These three components explained 99.4% of the variance in the original dataset (component 1 to 3 were 0.94, 0.05 and 0.01 respectively). The standard deviations decreased in order of components.

**Figure 3:**
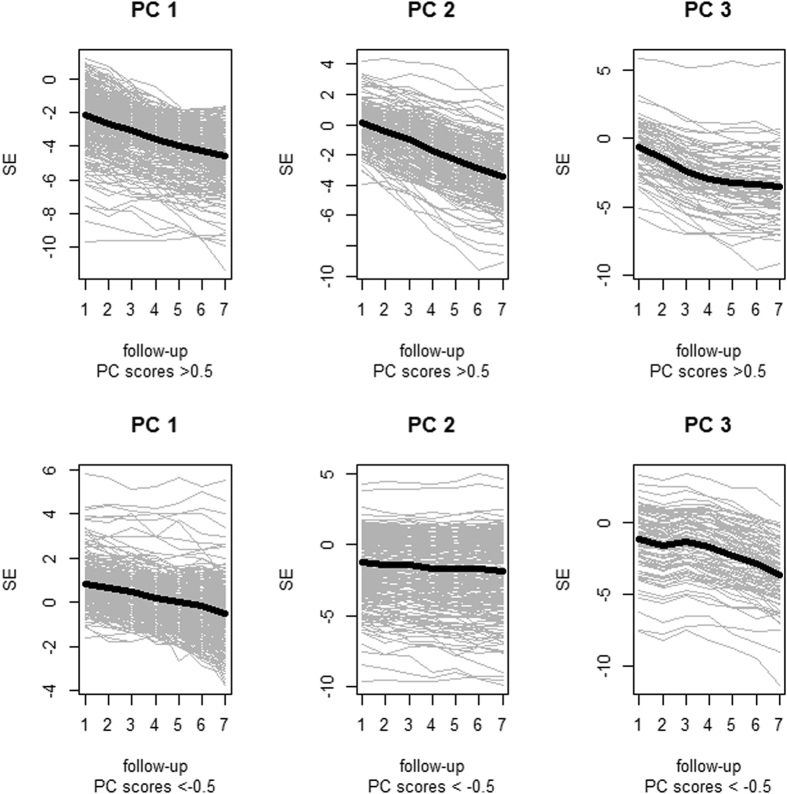
The patterns of different principle component scores in refraction progression (PC: principle component; SE: spherical equivalent).

From Fig. 3 we can appreciate a general impression of tendency across the three components with positive and negative component scores. In component 2, the acceleration appears to be faster with scores >0.5 than for scores < −0.5, but both remained in the same direction. Component 3 with scores < −0.5 showed a phase of plateau followed by a decrease in refraction, while component 3 with scores >0.5 initially decreased then reached a stable phase. This result suggests that component 3 is a combination of “myopia stabilization” and “late onset of refraction progress”. Therefore, component 3 with positive and negative scores can be separately analyzed. To better interpret the principle component, we named components 1 to 3 as “Average refraction”, “Acceleration” and “Stabilization or Late Onset” respectively.

Results of the analysis of associations between PCA scores and individual myopia-related risk factors, adjusting for baseline age and refraction, are presented in Table 3 and Table 4. The relationship between baseline refraction and component 2 was non-linear, therefore this non-linearity is adjusted using a natural cubic spline^20^ for the baseline SE, in the linear regression model of principle component 2. The regression coefficients suggested that children who had greater height change or weight change were positively associated with “Average refraction” (component 1), “Acceleration” (component 2) and “Stabilization” (component 3). Spending more time on reading and homework may accelerate the increase in refractive error. The greater severity of myopia of a parent, and having two myopic parents were also factors related to the “Acceleration” (component 2) of SE. Girls were found more likely to achieve stabilization (component 3 with scores >0). Outdoors activity time was negatively associated with component 3 at scores ≤0, suggesting that greater time spent outdoors was related to a later onset of myopia progression (component 3 with scores <0).

## Discussion

Hypothesis-independent methods were used in this study, featuring a cluster analysis and a principle component analysis. Three different groups of refraction progression were automatically identified to investigate the degrees of progression rate with cluster analysis. We deconstructed the process of refraction progression into three major components using PCA. Change of height and weight, near work time and parental myopia were associated with acceleration of refraction development, while the change of height and weight, and gender were predictors of refraction stabilization. Outdoor time was marginally significant for later onset of refraction progression in this young Chinese cohort.

The clustering to classify the different progressive groups was based on the methods that assign each observation to the nearest medoid, minimizing the sum of dissimilarities between the observations and the closest center^21^. Compared to the classification based on equal difference of refraction progression, the cluster analysis categorized observations with similar patterns according to the characteristics of data. For instance, the ranges of SE in the three groups classified by clustering were 0.20 to −0.27 D, −0.16 to −0.50 D and −0.36 to −1.11 D respectively, providing additional information of refraction progression to simple progression rates that some observations grow in different patterns despite similar amounts of annual progression rate. PCA is a novel method of identifying the different phases of refraction development. Existing models simulating refraction progression, the onset or cessation of myopia employ hypothesis-dependent methods of prediction^15^,^16^,^22^, while PCA deconstructs the process into independent components without simulating the refraction growth pathway. Therefore, the characteristics and possible mechanisms of individual components can be readily identified and analyzed.

According to previous longitudinal study data, refraction progression is associated with gender^23^,^24^,^25^ and time spent outdoors^11^,^25^, but not related to near work time^11^,^26^,^27^ or height^28^. Some studies investigated baseline refraction^11^,^29^ and time spent outdoors^25^,^30^ as determinants of myopia onset. The heritability of refraction is also widely documented^31^,^32^,^33^. Parental myopia, as a possible surrogate risk factor for genetic susceptibility, is found to be associated with higher prevalence of myopia^26^,^27^,^34^. In the GTES cohort, information on all these risk factors was collected and used for our current analysis. Clustering and PCA identified previously unrecognized risk factors, and revealed new information of the associations between risk factors and refraction progression.

Baseline SE, baseline age, maternal myopia and having two myopic parents were found significantly different among the three clusters. Baseline age tended to be younger in groups with greater amounts of progression, which is reiterated again in the linear regression model of principle component 2 and baseline age. Zhao *et al*. reported that myopic shift was associated with older age in a sample of children aged 5 to 15 years^8^. The association between baseline age and refraction progression in the present analysis was of opposite direction to that seen in Zhao *et al*., while remaining consistent with other studies demonstrating greater refraction progression in younger subjects^4^,^7^. The proportion of participants having two myopic parents is significantly higher in the group with fast progression of myopia, indicating that heredity has important effects on the growing direction of refraction. Myopia degree of father’s and mother’s appeared greatest in the fast progression cluster, second greatest in the slow progression cluster and least in the stable cluster. This suggests a likely dose-response relationship between parental myopia and patterns of refraction progression, as is indicated by other studies^27^,^35^.

In the acceleration component of refraction progression, change of height and weight, near work time, and parental myopia were statistically significant after controlling for baseline SE and age. Saw *et al*. found that reading increased the risk of myopia from cross-sectional data^36^, but in other longitudinal studies near work was neither associated with the onset nor the progression of myopia^11^,^28^,^37^. In the present analysis, longer near work time accelerated refraction progression. The disparity in these results may be explained by different phases of myopia being analyzed as an aggregate in previous studies, thereby omitting information of individuals’ progressions. Although having myopic parents may also interact with near work time, there is no evidence of this to date^11^,^27^,^31^. In the present study, paternal SE, maternal SE, and having two myopic parents did not demonstrate a linear relationship with near work time (data not shown). This agrees with previously established findings, that parental myopia is an independent risk factor of myopia. Our analysis indicates that parental refraction, having two myopic parents and near work greatly impact the acceleration of refractive progression. Further work is needed to explore the interaction between heredity and environment processes in the development of myopia.

The component representing stabilization of myopia was associated with gender as well as change of height and weight. In the Yip *et al*. study, girls tended to have earlier peak SE velocities and height velocities than boys, indicating that myopia progression is related to growth spurts^38^. The study also observed that girls have faster progression^8^,^9^. In view of our study results, it can be speculated that girls enter and complete refraction development earlier than boys following the patterns of growth in puberty. However, no evidence yet of associations between gender and age of stabilization has been revealed in other studies^22^. This association may warrant further investigation from future studies.

In our analysis, later onset of myopic shift showed a marginally significant relationship with time spent outdoors, according to the univariate regression model. Time outdoors did not produce a protective effect on myopia acceleration or influence myopic stabilization, but it was associated with the later onset of myopia shift. This is consistent with what Jones *et al*. found in an Australian cohort, which claimed that time outdoors was related to the timing of myopia onset rather than its progression^11^. This indicates that outdoor activity may play a role in postponing the onset of myopia but contributes little to impeding myopic acceleration, and that children may benefit from an intervention of outdoor activities before the onset of myopia.

Although the COMET study achieved a sample mean square error of 0.06 using the Gompertz function^22^, there is currently no evidence in the literature to validate the use of the fitting model. Therefore, the generalizability of the model to other data sets and populations remains unknown. In contrast, data-driven methods such as in our analysis, are hypothesis-independent and data adaptive, and offer an alternative that can be readily applied to different populations. The current limitation of our clustering and PCA analysis follows a lack of evaluation of specific ages for acceleration and stabilization of refraction. A more refined model addressing this problem should be explored in the future.

In conclusion, in the present study we investigated the variation in processes and the major components of refraction growth using data-driven methods. Younger age with greater myopia at baseline, parental myopia and longer time of near work were associated with accelerated progression of myopia. Furthermore, time spent outdoors was associated with the later onset of refraction progression.

## Methods

### Participants

The Guangzhou Twins Eye Study (GTES) is a longitudinal study including over 1200 pairs of twins and their parents or siblings living in Guangzhou, China^39^,^40^. The present study data was attained from the data of annual follow-up visits of GTES between 2006 and 2013. The baseline age of subjects ranged from 7 to 15 years. The GTES cohort was representative of the population in urban area of South China in terms of refractive status and schooling intensity. A total of 1279 first-born twin were included. Subjects with ocular media opacity, strabismus, orthokeratology treatment history, cataract surgery history, or loss of three consecutive visits were excluded (n = 642). Refraction of the right eyes of first-born twins was used. A total of 637 subjects were available for analysis.

### Measures

All the twins underwent autorefraction under cycloplegia at every visit. Cycloplegia was induced with 1% Cyclopentolate (1% Cyclogyl, Alcon Labs, Fort Wroth, Texas). Refraction was measured afterwards using an autorefractor (KR8800, Topcon Corp, Tokyo, Japan). An interviewer-administered questionnaire was used to collect information about demographic characteristics, near work activities and outdoor time for twins. Near work activity includes reading and homework on weekdays and weekends. Time of outdoor activities referred to the number of hours spent on morning exercise, travelling to school, returning back home and other activities such as walking or gardening on weekdays and weekends. The biological parents of the twins received autorefraction measurements in both eyes without cycloplegia. The sampling and methodology has been described in detail elsewhere^39^,^41^. Written informed consent was obtained from all the participants and their parents. The study obtained ethical committee approval from the Zhongshan University Ethical Review Board and Ethics Committee of Zhongshan Ophthalmic Center, and all examinations were conducted in accordance with the Tenets of the World Medical Association’s declaration of Helsinki.

### Statistical methods

Spherical equivalent (SE) was calculated as the sum of sphere and 1/2 cylinder. We adopted a partitioning clustering method to group similar growth patterns in first-born twins. The missing refraction, height and weight data (not more than two consecutive follow-ups) were imputed using the individual longitudinal regression imputation method^42^ to conduct the clustering. The partitioning clustering methods were based on the search for medoids among the observations of the dataset. The medoids were set to be SE minus the average of SE of 7 visits. Number of groups was set as 3. Principal component analysis (PCA) was performed to extract the main differences in how the refraction evolves over time, using a singular value decomposition of the centered data. Component scores were calculated using regression models. Ranges, frequency distributions, and consistency of all variables among related measurements were checked with data cleaning programs. The chi-square test was used to compare proportions among different groups. ANOVA tests were used to compare the differences in means of variables across different groups. The associations between PCA scores and individual myopia-related risk factors were assessed with independent linear regression models. Statistical analyses were performed with Stata 12.0 (Stata Corp., College Station, TX) and R (https://www.r-project.org/). All statistical tests were two-sided, with the α level being 5%.

## Additional Information

**How to cite this article**: Chen, Y. *et al*. Patterns in longitudinal growth of refraction in Southern Chinese children: cluster and principal component analysis. *Sci. Rep*. **6**, 37636; doi: 10.1038/srep37636 (2016).

**Publisher’s note:** Springer Nature remains neutral with regard to jurisdictional claims in published maps and institutional affiliations.

## Figures and Tables

**Table 1:** Characteristics of fist-born twins.

Characteristics	Mean	SD
N	637	—
Female(%)	51.3	—
Baseline age (year)	10.66	2.30
SE at baseline (diopter)	−0.52	1.97

SE: spherical equivalence, SD: standard deviation.

**Table 2:** Characteristics of the three cluster groups.

Group	1	2	3	*p*
Baseline SE (D)	−0.47 ± 2.16	−0.77 ± 2.00	−0.28 ± 1.50	0.0002*
Baseline age (year)	11.45 ± 2.42	10.41 ± 2.08	9.53 ± 1.79	<0.0001
Female (%)	52.67(148/281)	45.10(92/204)	53.29(81/152)	0.1840†
Change of height (mm)	8.60(22.66)	22.14(23.47)	29.75(25.3)	0.3665
Change of weight (kg)	11.54(15.24)	18.66(13.91)	22.02(10.35)	0.4571
Outdoor activity time (hour)	0.89(1.21)	0.97(1.14)	0.95(1.11)	0.8036
Near work time (hour)	4.02(2.30)	4.27(1.91)	4.00(1.86)	0.9565
Paternal refraction (D)	−0.56 ± 2.21	−1.03 ± 1.95	−1.42 ± 2.92	0.0050
Maternal refraction (D)	−0.75 ± 2.21	−1.03 ± 2.35	−2.06 ± 4.00	<0.0001
Two myopic parent (%)	10.23(27/264)	15.90(31/195)	22.67(34/150)	0.003†

Data was showed in form of mean ± sd or median (iqr).

One way ANOVA was used to compare the difference among the three groups unless otherwise noted.

^*^Adjusted for baseline age.

^†^Chi-square test.

**Table 3:** Linear regression for the component scores and related factors.

Parameter	Standardized Regression coefficient (β)			
	PC1	*p*	PC2	*p*
Model for baseline age and refraction				
Baseline age (year)	−0.13**	**<0.0001**	−0.42**	**<0.0001**
Baseline refraction(D)	−0.97**	**<0.0001**	0.21**	**<0.0001**
Univariate regression†				
Gender(female = 2)	0.02	0.2662	0.03	0.8288
Change of height (mm)	0.05**	**<0.0001**	0.03**	<0.0001
Change of weight (kg)	0.11**	**<0.0001**	0.04**	<0.0001
Outdoor time (hour)	−0.01	0.7290	0.01	0.8686
Near work time (hour)	0.03	0.0535	0.11*	**0.0198**
Paternal refraction (D)	−0.04*	**0.0244**	−0.06*	**0.0471**
Maternal refraction (D)	−0.05*	**0.0022**	−0.06*	**0.0122**
Two myopic parents	0.05*	**0.0183**	0.43*	**0.0187**

*p < 0.05.

**p < 0.001.

^†^Adjusting for baseline age and baseline refraction.

**Table 4:** Association between scores in principle component 3 and risk factors adjusting for baseline age and refraction.

Univariate regression†	Principle Component 3			
	Scores > 0	*p*	Scores ≤ 0	*p*
Gender(female = 2)	**0.71**	**0.0015**	0.25	0.2564
Change of height (mm)	**0.03**	**0.0014**	**0.04**	**0.0013**
Change of weight (kg)	**0.04**	**0.0014**	**0.03**	**0.0095**
Outdoor time (hour)	−0.08	0.5435	**−0.33**	**0.0422**
Near work time (hour)	0.04	0.6319	0.03	0.7540
Paternal refraction (D)	0.02	0.8003	−0.05	0.3713
Maternal refraction (D)	−0.02	0.6503	−0.03	0.5582
Two myopic parents	−0.51	0.4525	−0.12	0.6969

D: diopter.

^†^Adjusting for baseline age and baseline refraction.
